# Association study between *C10orf90* gene polymorphisms and colorectal cancer

**DOI:** 10.3389/fonc.2023.1192378

**Published:** 2023-07-31

**Authors:** Jian Song, Kaixuan Wang, Zhaowei Chen, Dunjing Zhong, Li Li, Liangliang Guo, Shuyong Yu

**Affiliations:** ^1^ Department of Gastroenterology, Southern University of Science and Technology Hospital, Shenzhen, Guangdong, China; ^2^ Department of Gastroenterology, The First Affiliated Hospital of Naval Medical University, Shanghai, China; ^3^ Department of Gastroenterology, Hainan Cancer Hospital, Haikou, Hainan, China; ^4^ Department of Gastrointestinal Surgery, Hainan Cancer Hospital, Haikou, Hainan, China

**Keywords:** colorectal cancer, *C10orf90*, gene polymorphisms, demographic characteristics, clinical features

## Abstract

**Background:**

Colorectal cancer (CRC) is the third most common malignant tumor in the world. The morbidity and mortality rates in Western countries have decreased, but they are still on the rise in China. *C10orf90* is associated with a variety of cancers, but the correlation between *C10orf90* and CRC is not yet known.

**Methods:**

A total of 1,339 subjects were randomly enrolled in our study. After extracting their DNA, three single-nucleotide polymorphisms (SNPs) of *C10orf90* were genotyped to analyze the potential relationship between these variants and CRC risk. PLINK software packages (version 1.07) were used to evaluate multiple genetic models by calculating the odds ratio (OR) and 95% confidence interval (95% CI). The best SNP–SNP interaction model was defined by the multifactor dimensionality reduction (MDR) analysis.

**Results:**

*C10orf90* rs12412320 was significantly associated with CRC risk (*p* = 0.006) and might be associated with the lower CRC risk (OR: 0.78; 95% CI: 0.65–0.93). The relationship of rs12412320 with lower CRC risk was found in people aged >60 years and ≤60 years, women, non-smokers, or non-drinkers. Rs11245008 in people aged ≤60 years and rs11245007 among men had a higher CRC susceptibility. Rs12412320 was related to the lower risk of advanced stages (III/IV stage), while rs11245007 might be associated with the higher risk of advanced stages (III/IV stage). Moreover, rs12412320 had the most significant relationship with the susceptibility to rectal cancer.

**Conclusion:**

This study is the first to report between *C10orf90* gene polymorphisms and CRC risk in Chinese people, which suggests that *C10orf90* rs12412320 might play a crucial role in preventing CRC occurrence.

## Introduction

Colorectal cancer (CRC) is the third (3.11%) most common malignant tumor in the world and the second (3.5%) leading cause of cancer death (1). Globally, there are approximately 1 million new CRC patients every year, and more than 915,880 patients die each year (1). The CRC incidence and mortality rates in China, Europe, and North America account for more than half of the world's CRC incidence and mortality, respectively (2). Most recently, the incidence rate of CRC has been increasing and has become the second most common malignant tumor in China, which seriously threatens the life and health of residents (3). In China, the survival rate of CRC in the recent 5 years is significantly lower than that of many developed countries (4, 5). Despite the high incidence and low survival rate of CRC in China, the pathogenesis of CRC remains unclear. Genetics and environment are the major factors in the development of CRC (6, 7). Previously, hyperlipidemia, obesity, alcohol consumption, and smoking were suggested to be risk factors, and other potential risk factors included hypertension, metabolic syndrome, dietary factors, sedentary behavior, and occupational exposure (8). Furthermore, genetic predisposition is one of the key risk factors in the development of CRC (9).

*C10orf90* (Chromosome 10 Open Reading Frame 90) is a protein coding gene and is known as the fragile-site associated tumor suppressor (FATS), which is also a regulator of the p53-p21 pathway (10). Studies have shown that in conjunctival melanoma, the deletion of the tumor suppressor gene *C10orf90* is related to the significantly reduced metastasis-free survival of tumor patients (11). In addition, *C10orf90* is a target gene of p53, and its overexpression can inhibit tumorigenicity *in vivo*, which is related to anti-tumor activity (12). FATS is an E2- and E3-independent ubiquitin ligase for promoting p53 stability and activation in response to DNA damage (13). The expression of *C10orf90* gene is downregulated or silenced in many cancers, and it is related to non-small cell lung cancer, breast cancer, and others (14, 15). Furthermore, *C10orf90* variants have been reported to be associated with the risk of various cancers, including breast cancer (16) and conjunctival melanomas (11). However, whether the genetic variants in *C10orf90* may modulate CRC susceptibility remain unknown.

Single-nucleotide polymorphism (SNP) in the coding regions of genes may affect protein function. Here, three polymorphisms in the exon region of *C10orf90* were genotyped to explore the relationship with CRC susceptibility in the Chinese Han population and to correlate these with demographic characteristics and clinical features.

## Methods

### Subjects

In this study, a total of 666 CRC patients at Hainan Province Cancer Hospital from August 2020 to December 2022 were randomly enrolled in the case group. A total of 673 healthy adults form the control group; they were from the same hospital during the same period without a history of cancer and chronic or severe diseases. The selection criteria of patients complied with the “Guidelines for diagnosis and treatment of colorectal cancer (2021 CSCO)” (17), and all patients were independent of each other. Patients suffering from inflammation, renal dysfunction, digestive system disease, and other chronic or endocrine disease, and who have been receiving any anti-cancer drugs or treatments were excluded. Demographic and clinical information of all subjects were gathered through standardized questionnaires and medical records, which include age, sex, smoking status, drinking status, body mass index (BMI), cancer stage, lymph node metastasis status, cancer style, carcinoembryonic antigen (CEA), alpha-fetoprotein (AFP), and cancer antigen-199 (CA199). The study was approved by the ethical committee of Hainan Province Cancer Hospital, and informed consent forms were signed by all subjects before the study, according to the Helsinki Declaration.

### DNA extraction and SNP genotyping

Three SNPs (rs12412320, rs11245007, and rs11245008) in *C10orf90* were selected for the study of their potential role in the risk of CRC based on a minor allele frequency (MAF) > 0.05 through the 1000 Genome Project. The potential biological functions of these loci were predicted through bioinformatics databases, including dbSNP, RegulomeDB, VannoPortal, and HaploReg v4.2.

Genomic DNA was extracted from peripheral blood samples (5 mL) of each subject using the Whole Blood Genomic DNA Isolation Kit (Xi’an Gold Mag Biotechnology, Xi'an, China). DNA was stored together with EDTA in a tube at −80°C. DNA concentrations were measured using NanoDrop 2000 (Ultra-fine ultraviolet spectrophotometer, Thermo, Waltham, MA, USA). SNP genotyping with a standard protocol was carried out using Agena MassARRAY RS1000. Agena Typer Software version 4.0 was used for data management.

### Data analysis

Independent samples *t*-test and Chi-square test were used to assess the differences in demographic characteristics of the study participants. We used Fisher’s test to evaluate the Hardy–Weinberg equilibrium (HWE) of each SNP in the subjects. Odds ratio (OR) and 95% confidence interval (95% CI) were assessed to estimate the correlations of SNPs and CRC risk using logistic regression analysis. PLINK software packages (version 1.07) were used to evaluate multiple genetic models (allele model, genotype model, dominant model, recessive model, and additive model). Statistical analysis was performed using Microsoft Excel and SPSS 17.0 statistical packages (SPSS, Chicago, IL). A two-tailed *p* < 0.05 was considered statistically significant, and a Bonferroni-corrected *p* < 0.05/3 was considered significant. In addition, we used the multifactor dimensionality reduction (MDR) analysis to identify the best SNP–SNP interaction model.

## Results

### Characteristics of subjects

There were 1,339 subjects in this study, namely, 666 CRC patients (age: 60.02 ± 11.28 years) and 673 healthy controls (age: 59.53 ± 9.63 years). **Table 1** shows the relevant characteristics of all subjects including the case group and the control group. It can be seen that there are no statistical differences between CRC patients and healthy controls in these indexes such as age (*p* = 0.391), sex (*p* = 0.698), smoking (*p* = 0.372), and drinking (*p* = 0.438). There was a significant difference in BMI between CRC patients and healthy controls (*p* < 0.001).

**Table 1:** Characteristics of colorectal cancer patients and healthy controls.

variable	Patients ( n=666 )	Controls ( n=673 )	*p*
Age (years)	60.017 ± 11.275	59.525 ± 9.634	0.391
> 60	350 (52.6%)	377 (56.0%)	
≤ 60	316 (47.4%)	296 (44.0%)	
Sex			0.698
male	383 (57.5%)	395 (58.7%)	
female	283 (42.5%)	278 (41.3%)	
Smoking Status			0.372
Yes	257 (38.6%)	276 (41.0%)	
No	409 (61.4%)	397 (59.0%)	
Drinking Status			0.438
Yes	270 (40.5%)	287 (42.6%)	
No	396 (59.5%)	386 (57.4%)	
BMI (kg/m^2^)	22.441 ± 3.355	24.215 ± 3.364	***p* < 0.001**
> 24	155 (23.3%)	214 (31.8%)	
≤ 24	305 (45.8%)	200 (29.7%)	
Missing	252 (37.8%)	213 (31.6%)	
Stage			
I/II	94 (14.1%)		
III/IV	212 (31.8%)		
Missing	360 (54.1%)		
Lymph node metastasis			
Yes	233 (35.0%)		
No	133 (20.0%)		
Missing	300 (45.0%)		
Cancer Style			
Colon cancer	293 (44%)		
Rectal cancer	351 (52.7%)		
Missing	22 (3.3%)		

BMI, body mass index.

p values were calculated using Chi-square test or T test, two sided.

Bold indicates statistical significance at P < 0.05.

### Relationship between *C10orf90* SNPs and CRC risk

**Figure 1:**
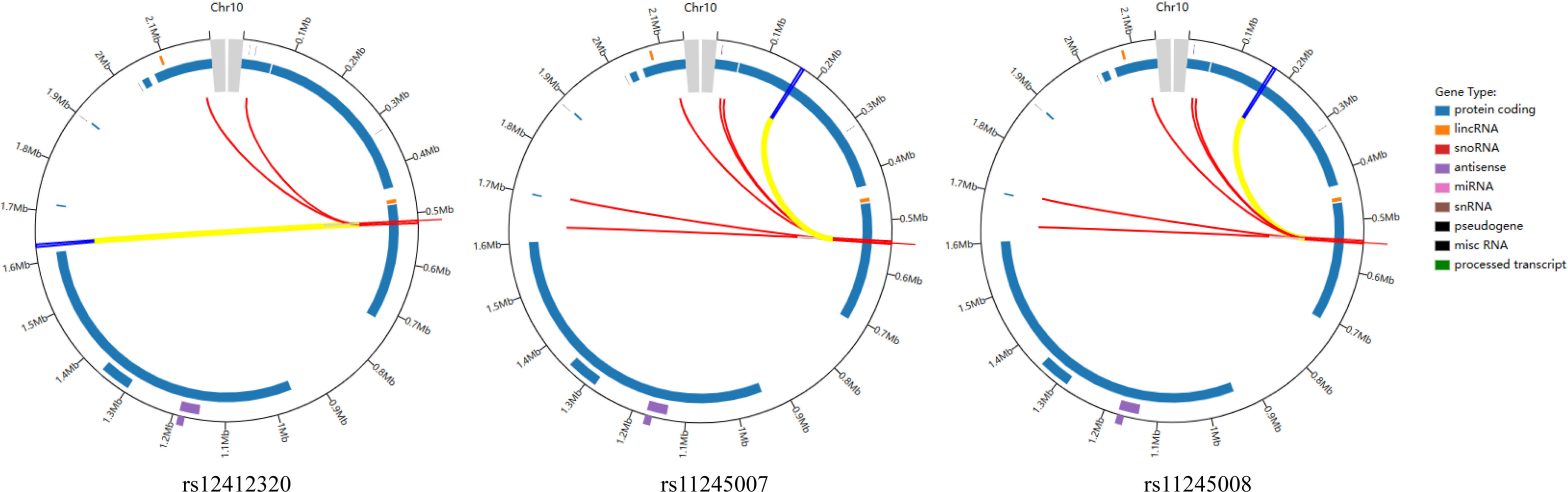
Virtual 4C circular plot for the most significant Hi–C interactions between the variant locus and the target regions.

The relationship between SNPs of *C10orf90* and CRC risk is listed in **Table 2**. All SNPs were missense variants. All SNPs of *C10orf90* complied with the Hardy–Weinberg equilibrium (*p* > 0.05). The MAF of each SNP was above 5% in the Chinese Han population. *C10orf90* rs12412320 was significantly associated with CRC risk (*p* = 0.006) and might be associated with the lower CRC risk (OR: 0.78; 95% CI: 0.65–0.93). Bioinformatics analysis found that these SNPs may be involved in promoter/enhancer histone marks, and protein-bound motifs changed the binding of transcription factors (TFs) and the action of DNase. **Figure 1** shows the most significant Hi–C interactions between the variant locus and the target regions.

**Table 2:** The based information of selected SNPs in *C10orf90* and the association with the risk of colorectal cancer in the allele model.

SNP	Chromosome	Alleles A / B	dbSNP func annot	MAF		p HWE	OR (95% CI)	p *	RegulomeDB	HaploReg v4.2
				Case	Control					
rs12412320	10:126461527	T/G	Missense D (Asp) > E (Glu)	0.205	0.249	0.758	0.78 ( 0.65 - 0.93 )	0.006*	TF binding or DNase peak	Enhancer histone marks, Motifs changed
rs11245007	10:126504416	T/C	Missense D (Asp) > N (Asn)	0.480	0.452	0.436	1.12 ( 0.96 - 1.31 )	0.134	TF binding + any motif + DNase peak	Promoter histone marks, Enhancer histone marks, DNAse, Proteins bound, Motifs changed
rs11245008	10:126504799	T/C	Missense R (Arg) > L (Leu)	0.137	0.121	0.145	1.16 ( 0.92 - 1.45 )	0.209	TF binding + any motif + DNase peak	Enhancer histone marks, DNAse, Motifs changed

SNP, Single nucleotide polymorphism; MAF, Minor allele frequency; HWE, Hardy-Weinberg equilibrium; OR, Odds ratio; 95% CI, 95% confidence interval.

p values of Hardy-Weinberg equilibrium were calculated using Chi-square test.

p values were calculated by two sided Chi-square test, and * p < 0.05 was considered statistical significance.

Bold p means that the data is statistically significant after Bonferroni correction (p < 0.05/3).

dbSNP (https://www.ncbi.nlm.nih.gov/snp/), RegulomeDB (https://regulome.stanford.edu/regulome-search/) and HaploReg v4.2 (https://pubs.broadinstitute.org/mammals/haploreg/haploreg.php).

**Table 3** shows the relationship between CRC risk and the different genetic models of *C10orf90* polymorphisms in the overall analysis. Logistic regression analysis showed that, whether corrected or not, there were significant differences in the correlation between SNPs of *C10orf90* rs12412320 and the risk of CRC. Among them, three allele models of rs12412320 (Heterozygous: *p* = 0.003, OR: 0.70, 95% CI: 0.56, 0.89; Dominant: *p* = 0.002, OR:0.71, 95% CI: 0.57, 0.88; Additive: *p* = 0.005, OR: 0.77, 95% CI: 0.64, 0.93, adjusted) were significantly correlated with the risk of CRC. The protective significance of rs12412320 for CRC occurrence still existed after Bonferroni multiple correction (*p* < 0.05/3).

**Table 3:** Selected variants in C10orf90 associated with the risk of colorectal cancer.

SNP	Model	Genotype	Control	Case	Without adjusted		With adjusted	
					OR (95% CI)	p	OR (95% CI)	p
rs12412320	Genotype	G/G	377 (56.1%)	427 (64.1%)	1			
		G/T	255 (38.0%)	205 (30.8%)	0.71 (0.56, 0.89)	0.004*	0.70 (0.56, 0.89)	0.003*
		T/T	40 (6.0%)	34 (5.1%)	0.75 (0.46, 1.21)	0.239	0.74 (0.46, 1.19)	0.211
	Dominant	G/G	377 (56.1%)	427 (64.1%)	1			
		G/T-T/T	295 (43.9%)	239 (35.9%)	0.72 (0.57, 0.89)	0.003*	0.71 (0.57, 0.88)	0.002*
	Recessive	G/G-G/T	632 (94.0%)	632 (94.9%)	1			
		T/T	40 (6.0%)	34 (5.1%)	0.85 (0.53, 1.36)	0.498	0.84 (0.52, 1.34)	0.463
	Additive	---	---	---	0.78 (0.65, 0.93)	0.007*	0.77 (0.64, 0.93)	0.005*
rs11245007	Genotype	C/C	207 (30.9%)	192 (28.8%)	1			
		C/T	322 (48.0%)	308 (46.2%)	1.03 (0.80, 1.33)	0.810	1.04 (0.81, 1.34)	0.747
		T/T	142 (21.2%)	166 (24.9%)	1.26 (0.94, 1.70)	0.128	1.27 (0.94, 1.72)	0.113
	Dominant	C/C	207 (30.9%)	192 (28.8%)	1			
		C/T-T/T	464 (69.2%)	474 (71.2%)	1.10 (0.87, 1.39)	0.420	1.13 (0.88, 1.41)	0.375
	Recessive	C/C-C/T	529 (78.8%)	500 (75.1%)	1			
		T/T	142 (21.2%)	166 (24.9%)	1.24 (0.96, 1.60)	0.103	1.24 (0.96, 1.61)	0.098
	Additive	---	---	---	1.12 (0.96, 1.30)	0.144	1.12 (0.97, 1.30)	0.127
rs11245008	Genotype	C/C	524 (77.9%)	497 (74.6%)	1			
		C/T	135 (20.1%)	155 (23.3%)	1.21 (0.93, 1.57)	0.152	1.21 (0.93, 1.58)	0.148
		T/T	14 (2.1%)	14 (2.1%)	1.05 (0.50, 2.23)	0.890	1.03 (0.49, 2.20)	0.931
	Dominant	C/C	524 (77.9%)	497 (74.6%)	1			
		C/T-T/T	149 (22.1%)	169 (25.4%)	1.20 (0.93, 1.54)	0.164	1.20 (0.93, 1.54)	0.164
	Recessive	C/C-C/T	659 (97.9%)	652 (97.9%)	1			
		T/T	14 (2.1%)	14 (2.1%)	1.01 (0.48, 2.14)	0.978	0.99 (0.47, 2.11)	0.987
	Additive	---	---	---	1.15 (0.92, 1.44)	0.218	1.15 (0.92, 1.44)	0.223

SNP, Single nucleotide polymorphis; OR, Odds ratio; 95% CI, 95% confidence interval.

p values were calculated by logistic regression analysis without and with adjusted by sex, age, smoking, and drinking

*p < 0.05 was considered statistical significance.

Bold p means that the data is statistically significant after Bonferroni correction (p < 0.05/3).

### *C10orf90* SNPs associated with CRC risk in the stratified analysis

**Figure 2:**
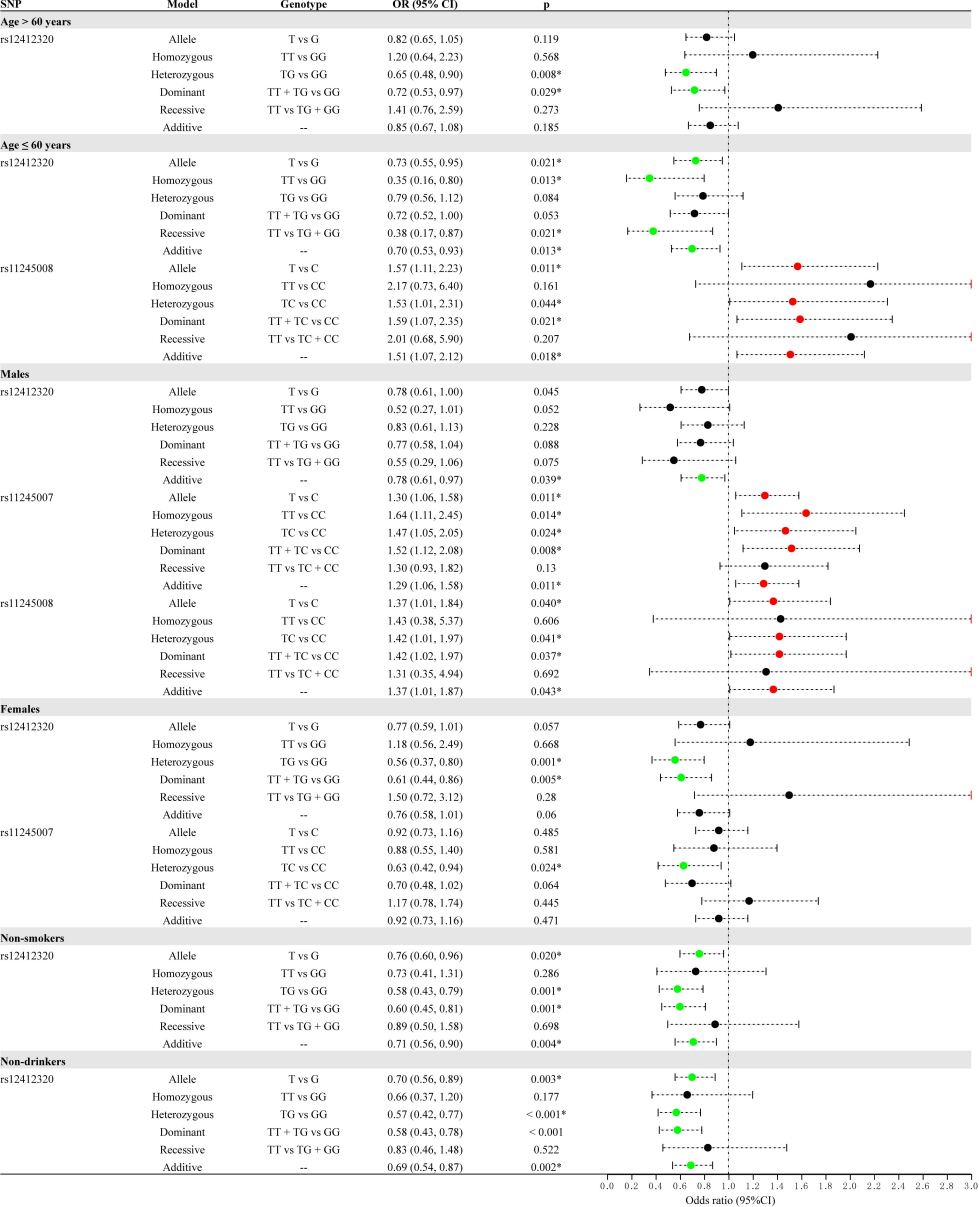
Forest map for the stratification analysis by demographic characteristics (age, sex, smoking, and drinking) for the association between *C10orf90* variants and colorectal cancer (CRC) risk.

To explore the relationship of three SNPs with CRC, we performed the subgroup stratification analysis by demographic characteristics (age, sex, smoking, drinking, and BMI), as shown in **Supplementary Table S1** and **Figure 2**. After Bonferroni multiple correction, the relationship of rs12412320 in people aged >60 years (*p* = 0.008, OR: 0.65) and ≤60 years (*p* = 0.013, OR: 0.35, and *p* = 0.013, OR: 0.70) and that of rs11245008 in people aged ≤60 years (*p* = 0.011, OR: 1.57) were also remarkable. In the sex-stratified analysis, rs12412320 (*p* = 0.001, OR: 0.56; and *p* = 0.005, OR: 0.61) had a lower CRC risk in women, whereas rs11245007 (*p* = 0.011, OR: 1.30; *p* = 0.008, OR: 1.52; and *p* = 0.011, OR: 1.29) had a higher CRC susceptibility among men after Bonferroni multiple correction. After Bonferroni multiple correction, *C10orf90* rs12412320 was also significantly associated with CRC in non-smokers (*p* = 0.001, OR: 0.58; *p* = 0.001, OR: 0.60; and *p* = 0.004, OR: 0.71) and non-drinkers (*p* = 0.003, OR: 0.70; *p* < 0.001, OR: 0.57; *p* < 0.001, OR: 0.58; and *p* = 0.002, OR: 0.69).

**Figure 3:**
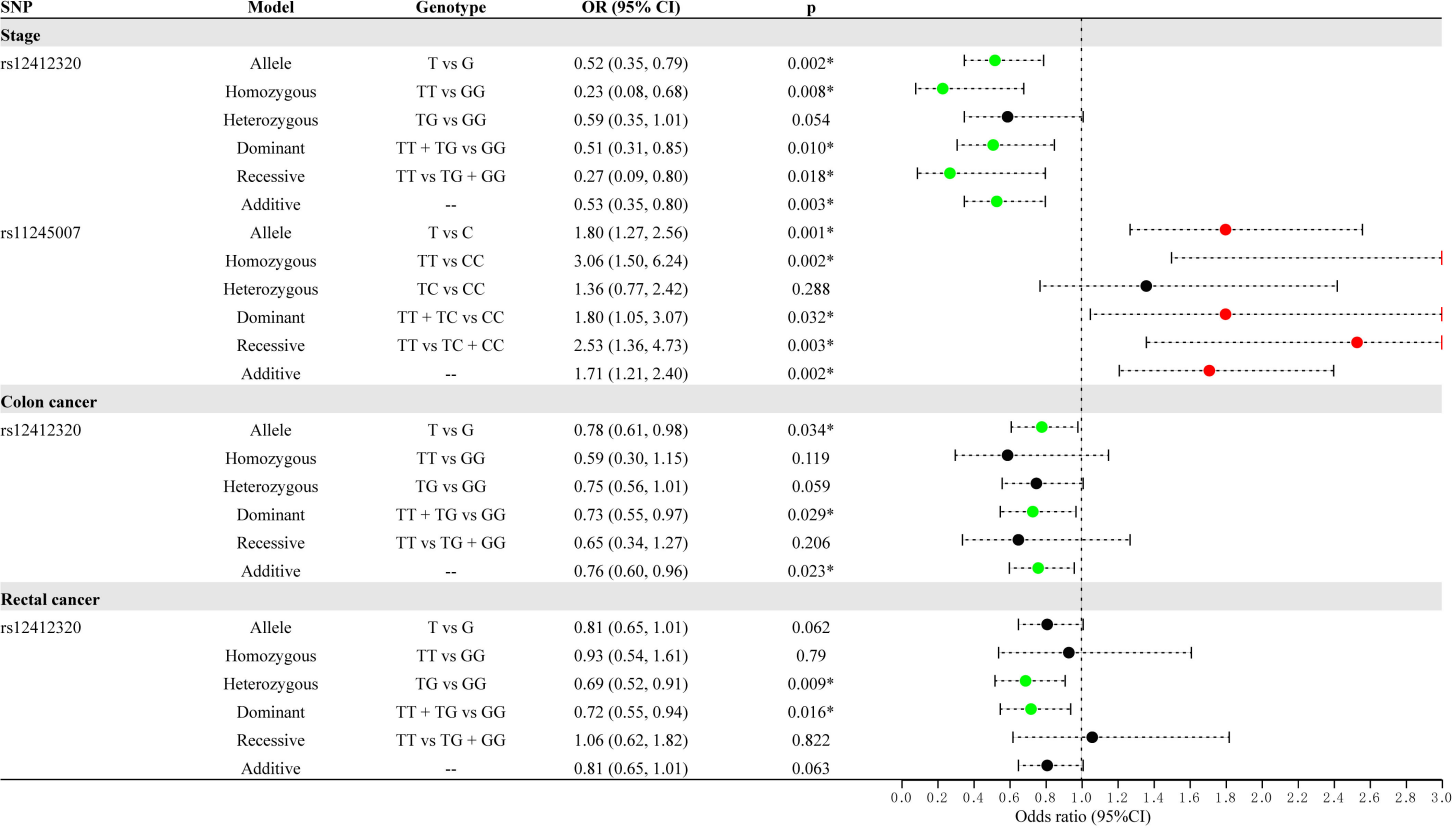
Forest map for the stratification analysis by clinical features (cancer type and stage) for the association between *C10orf90* variants and colorectal cancer (CRC) risk.

Stratified analysis by clinical features (stage, lymph node metastasis, and cancer style) for the association between *C10orf90* variants and the risk of CRC is displayed in **Supplementary Table S2** and **Figure 3**. After Bonferroni multiple correction, rs12412320 (*p* = 0.002, OR: 0.52; *p* = 0.008, OR: 0.23; *p* = 0.010, OR: 0.51; and *p* = 0.003, OR: 0.53) was related to the lower risk of advanced stages (III/IV stage), while rs11245007 (*p* = 0.001, OR: 1.80; *p* = 0.002, OR: 3.06; *p* = 0.003, OR: 2.53; and *p* = 0.002, OR: 1.71) might be associated with the higher risk of advanced stages (III/IV stage). Rs12412320 (*p* = 0.009, OR: 0.69; and *p* = 0.016, OR: 0.72) had the most significant relationship with the susceptibility of rectal cancer after Bonferroni multiple correction. Moreover, rs12412320 was associated with the risk of colon cancer, but no significance was found after Bonferroni multiple correction.

### MDR analysis for *C10orf90* variants

**Figure 4:**
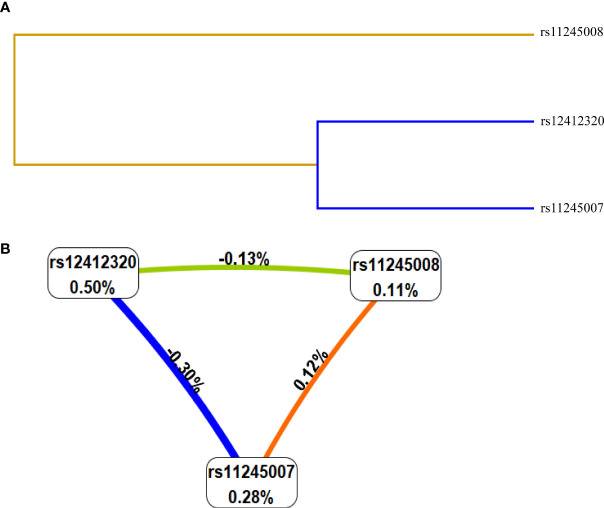
Interaction map among single-nucleotide polymorphisms (SNPs) of genes on the risk of colorectal cancer (CRC). Values in nodes represent the information gain (IGs) of individual attribute (main effects). Values between nodes are IGs of each pair of attributes (interaction effects). SNP-SNP interaction dendrogram **(A)** and Fruchterman-Reingold **(B)**.

Then, the relationship between the interaction of *C10orf90* SNPs and CRC risk was analyzed by the MDR method. The results of the MDR model analysis of the SNP–SNP interactions are demonstrated in **Table 4** and **Figure 4**. The dendrogram (**Figure 4A**) shows that loci with strong interactions were located very close to each other on the branches, while loci with weak interactions were far apart from each other. The most significant single-locus model was rs12412320 [testing accuracy: 0.5338, *p* = 0.0077, cross-validation consistency (CVC): 10/10] with an information gain of 0.50% (**Figure 4B**); the best two-locus models were rs12412320 and rs11245008 (testing accuracy: 0.5308, *p* = 0.0041, CVC: 6/10); and the best three-locus models were rs12412320, rs11245007, and rs11245008 (testing accuracy: 0.5300, *p* = 0.0007, CVC: 10/10), which is the best SNP–SNP interaction model. Therefore, the impact of the three candidate SNPs on the risk of CRC may be interdependent.

**Table 4:** Summary of SNP – SNP interactions on the risk of colorectal cancer analyzed through MDR method.

Model	Training Bal. Acc. ( % )	Testing Bal. Acc. ( % )	CVC	OR (95% CI)	*p*
rs12412320	0.54	0.54	10/10	1.40 ( 1.13, 1.75 )	**0.0025**
rs12412320, rs11245008	0.54	0.53	5/10	1.42 ( 1.14, 1.77 )	**0.0015**
rs12412320, rs11245007, rs11245008	0.55	0.53	10/10	1.48 ( 1.20, 1.84 )	**0.0003**

MDR, multifactor dimensionality reduction; Bal. Acc., balanced accuracy; CVC, cross–validation consistency; OR, odds ratio; CI, confidence interval.

p values were calculated using Chi-square test, two sided.

Bold indicates statistical significance at P < 0.05.

## Discussion

The multi-disciplinary approach that combines genetics, immunology, and chemotherapy has the potential to revolutionize the treatment of CRC and other types of cancer as well. One of the major challenges in cancer treatment is the heterogeneity of tumors, which can make it difficult to develop effective therapies. However, by understanding the molecular mechanisms underlying cancer and the role of the immune system in cancer development and progression, researchers can develop personalized treatment approaches that target the specific characteristics of each patient’s tumor (18). Genetic factors are important influencing factors of CRC. Research shows that approximately 5% of CRC is caused by chromosomal variation, which is hereditary (19). Previous studies have reported many loci associated with the risk of CRC (20–22). However, the specific molecular mechanism of CRC has not been fully understood. There are still a large number of loci that may affect the risk of CRC that have not been reported. Therefore, further exploring the relationship between gene SNPs and CRC risk is much more significant and useful for the specific diagnosis on CRC. As a tumor suppressor associated with fragile sites, *C10orf90* is involved in DNA damage-induced carcinogenesis. *C10orf90* overexpression significantly enhances the sensitivity of non-small cell lung cancer (NSCLC) cells to cisplatin, and is related to the overall survival rate (23). In this study, we analyzed the association between genetic polymorphisms of *C10orf90* and the risk of CRC in 1,339 Chinese people. The results displayed that the genetic polymorphisms of *C10orf90* were significantly associated with the risk of CRC, especially SNP rs12412320. Here, we had reported for the first time that *C10orf90* rs12412320 was associated with a reduced risk of CRC in the Chinese Han population. There are currently few reports on this locus. Bioinformatics analysis revealed that these SNPs may be related to promoter/enhancer histone marks, and protein-bound motifs changed the binding of TFs and the action of DNase. This indicates that *C10orf90* rs12412320 may affect the risk of CRC by affecting the expression of the gene.

At present, it is universally recognized that the occurrence of CRC is related to immutable risk factors, including age, sex, genetic factors, environment, and lifestyle (6, 7, 24). CRC usually appears after 50 years of age, and the incidence rate of CRC in women is low, usually accounting for one-third of the total incidence rate (25). The combination of tobacco and alcohol increases the risk of cancer. Smoking increases the susceptibility to CRC in a dose-dependent manner with intensity and duration (26). Alcohol consumption and obesity are considered modifiable risk factors for CRC (27, 28). The stratification analysis was explored for the effect of demographic characteristics (age, sex, smoking, drinking, and BMI) on the relationship of three SNPs with CRC. After Bonferroni multiple correction, the relationship of rs12412320 with lower CRC risk was found in people aged >60 years and ≤60 years, women, non-smokers, or non-drinkers. Some studies have suggested that smoking, which created a hypoxic microenvironment that was quite common in solid tumors, might cooperate with genetic polymorphism to produce a superimposed effect on the progression of CRC (29). Previously, possible interactions between GWAS-identified CRC susceptibility SNPs and alcohol consumption were investigated, and genetic polymorphisms were associated with increased risk of CRC among ever drinkers and higher-level alcohol drinkers, suggesting that alcohol consumption could be a possible effect modifier (30). Our study found that *C10orf90* rs12412320 was associated with a reduced risk of CRC overall. The stratification analysis showed that *C10orf90* rs12412320 was also significantly associated with CRC in non-smokers and non-drinkers, but not smokers and drinkers. These results suggested that not smoking or not drinking was found to reduce the likelihood of CRC risk among the population who carried *C10orf90* rs12412320-T allele. Moreover, rs11245008 in people aged ≤60 years and rs11245007 among men had a higher CRC susceptibility after Bonferroni multiple correction. Functional analysis demonstrated that rs11245007, a functional variant of *C10orf90*, can modulate p53 activation, resulting from the more pronounced polyubiquitination of p53 by rs11245007-T (mutant allele) (16). Song et al. showed that rs11245007 played a vital role in preventing the occurrence of breast cancer (16). Here, rs11245007 can increase the risk of CRC in men, which is opposite to its role in breast cancer. It may be caused by the different pathogenesis of various diseases, tumor heterogeneity, and so on. Our study provides evidence to clarify that the pathogenic effect on CRC may be partially attributed to the interaction between *C10orf90* variants and age, sex, smoking, and alcohol consumption.

The clinical characteristics of CRC patients are related to prognosis, and the complex interaction between staging, metastasis, and genetic factors plays a role in guiding prognosis, risk stratification, and adjuvant treatment of CRC (31, 32). In the study, stratified analysis by clinical features (stage, lymph node metastasis, and cancer style) for the association between *C10orf90* variants and the risk of CRC was investigated. After Bonferroni multiple correction, rs12412320 was related to the lower risk of advanced stages (III/IV stage), while rs11245007 might be associated with the higher risk of advanced stages (III/IV stage). Moreover, rs12412320 had the most significant relationship with the susceptibility of rectal cancer after Bonferroni multiple correction.

Unavoidably, this study has several limitations. Firstly, the study was conducted in a single hospital in Hainan Province, China, which limits the generalizability of the results to other populations. Secondly, because a proportion of the samples lack information on environmental factors (such as diet, physical activity, and environmental factors) and because of the relatively small sample size, our study did not explain the role of the interaction between *C10orf90* variants and environmental factors on CRC risk. In the future, we would like to increase the sample size and complete the environmental factors to evaluate the relationship and to verify our findings. Thirdly, only three SNPs of *C10orf90* were studied in this study, and other genetic variants that may play a role in CRC susceptibility were not investigated. Experimental design will continue to explore the correlation of other loci on this gene with CRC risk in the future. Fourthly, the mechanism of these SNPs is only predicted through bioinformatics analysis; therefore, functional experiments are needed to further explore the function of *C10orf90* loci in CRC etiology. Fifthly, CRC patients’ tissues and normal tissues had not been explored in protein expression studies. In subsequent research, we plan to collect enough CRC patients’ tissues and normal tissues to examine them *via* immunohistochemistry (IHC) using protein expression studies and to conduct functional research of these SNPs in CRC.

## Conclusion

In summary, this study is the first to report the relationship between *C10orf90* gene polymorphisms and CRC risk in Chinese people, which suggests that *C10orf90* rs12412320 might play a crucial role in preventing CRC occurrence. It provides the foundation for the study on the mechanism of *C10orf90* in CRC and supplies the basis for personalized treatment of CRC patients.

## Data availability statement

The original contributions presented in the study are included in the article/**Supplementary Material**. Further inquiries can be directed to the corresponding author.

## Ethics statement

The studies involving human participants were reviewed and approved by Hainan Cancer Hospital. The patients/participants provided their written informed consent to participate in this study.

## Author contributions

JS and KW wrote the manuscript, ZC and DZ processed and analyzed the results, LL and LG prepared **Figure 1** and collected data, and SY designed the research ideas and plans. All the authors reviewed the manuscript. All authors contributed to the article and approved the submitted version.
