# Supplementary material for: Association study between C10orf90 gene polymorphisms and colorectal cancer
Source: Front Oncol. 2023 Jul 31;13:1192378. doi: 10.3389/fonc.2023.1192378 (PMC10425599; doi:10.3389/fonc.2023.1192378)
Supplement: Supplementary file 1 [file Table_1.docx]

**Table S1: Stratified analysis by demographic characteristics for the association between *C10orf90* variants and the risk of colorectal cancer.**

| SNP | Model | Genotype | OR (95% CI) | *p* | OR (95% CI) | *p* |
| --- | --- | --- | --- | --- | --- | --- |
| **Age** |  |  | **> 60 years** | | **≤ 60 years** | |
| rs12412320 | Allele | T vs G | 0.82 (0.65, 1.05) | 0.119 | 0.73 (0.55, 0.95) | 0.021* |
|  | Homozygous | TT vs GG | 1.20 (0.64, 2.23) | 0.568 | 0.35 (0.16, 0.80) | **0.013*** |
|  | Heterozygous | TG vs GG | 0.65 (0.48, 0.90) | **0.008*** | 0.79 (0.56, 1.12) | 0.084 |
|  | Dominant | TT + TG vs GG | 0.72 (0.53, 0.97) | 0.029* | 0.72 (0.52, 1.00) | 0.053 |
|  | Recessive | TT vs TG + GG | 1.41 (0.76, 2.59) | 0.273 | 0.38 (0.17, 0.87) | 0.021* |
|  | Additive | -- | 0.85 (0.67, 1.08) | 0.185 | 0.70 (0.53, 0.93) | **0.013*** |
| rs11245007 | Allele | T vs C | 1.18 (0.96, 1.45) | 0.114 | 1.06 (0.84, 1.32) | 0.632 |
|  | Homozygous | TT vs CC | 1.37 (0.91, 2.06) | 0.133 | 1.18 (0.76, 1.85) | 0.462 |
|  | Heterozygous | TC vs CC | 1.01 (0.72, 1.43) | 0.936 | 1.10 (0.76, 1.61) | 0.612 |
|  | Dominant | TT + TC vs CC | 1.12 (0.81, 1.54) | 0.498 | 1.13 (0.79, 1.61) | 0.504 |
|  | Recessive | TT vs TC + CC | 1.36 (0.95, 1.92) | 0.089 | 1.11 (0.76, 1.63) | 0.581 |
|  | Additive | -- | 1.16 (0.95, 1.42) | 0.157 | 1.09 (0.87, 1.36) | 0.457 |
| rs11245008 | Allele | T vs C | 0.92 (0.68, 1.25) | 0.606 | 1.57 (1.11, 2.23) | **0.011*** |
|  | Homozygous | TT vs CC | 0.35 (0.09, 1.33) | 0.123 | 2.17 (0.73, 6.40) | 0.161 |
|  | Heterozygous | TC vs CC | 1.07 (0.75, 1.51) | 0.719 | 1.53 (1.01, 2.31) | 0.044* |
|  | Dominant | TT + TC vs CC | 1.00 (0.71, 1.40) | 0.982 | 1.59 (1.07, 2.35) | 0.021* |
|  | Recessive | TT vs TC + CC | 0.35 (0.09, 1.31) | 0.118 | 2.01 (0.68, 5.90) | 0.207 |
|  | Additive | -- | 0.93 (0.69, 1.26) | 0.650 | 1.51 (1.07, 2.12) | 0.018* |
| **Sex** |  |  | **Male** | | **Female** | |
| rs12412320 | Allele | T vs G | 0.78 (0.61, 1.00) | 0.045 | 0.77 (0.59, 1.01) | 0.057 |
|  | Homozygous | TT vs GG | 0.52 (0.27, 1.01) | 0.052 | 1.18 (0.56, 2.49) | 0.668 |
|  | Heterozygous | TG vs GG | 0.83 (0.61, 1.13) | 0.228 | 0.56 (0.37, 0.80) | **0.001*** |
|  | Dominant | TT + TG vs GG | 0.77 (0.58, 1.04) | 0.088 | 0.61 (0.44, 0.86) | **0.005*** |
|  | Recessive | TT vs TG + GG | 0.55 (0.29, 1.06) | 0.075 | 1.50 (0.72, 3.12) | 0.280 |
|  | Additive | -- | 0.78 (0.61, 0.97) | 0.039* | 0.76 (0.58, 1.01) | 0.060 |
| rs11245007 | Allele | T vs C | 1.30 (1.06, 1.58) | **0.011*** | 0.92 (0.73, 1.16) | 0.485 |
|  | Homozygous | TT vs CC | 1.64 (1.11, 2.45) | 0.014* | 0.88 (0.55, 1.40) | 0.581 |
|  | Heterozygous | TC vs CC | 1.47 (1.05, 2.05) | 0.024* | 0.63 (0.42, 0.94) | 0.024* |
|  | Dominant | TT + TC vs CC | 1.52 (1.12, 2.08) | **0.008*** | 0.70 (0.48, 1.02) | 0.064 |
|  | Recessive | TT vs TC + CC | 1.30 (0.93, 1.82) | 0.130 | 1.17 (0.78, 1.74) | 0.445 |
|  | Additive | -- | 1.29 (1.06, 1.58) | **0.011*** | 0.92 (0.73, 1.16) | 0.471 |
| rs11245008 | Allele | T vs C | 1.37 (1.01, 1.84) | 0.040* | 1.04 (0.74, 1.45) | 0.638 |
|  | Homozygous | TT vs CC | 1.43 (0.38, 5.37) | 0.606 | 0.81 (0.32, 2.04) | 0.651 |
|  | Heterozygous | TC vs CC | 1.42 (1.01, 1.97) | 0.041* | 0.97 (0.63, 1.50) | 0.905 |
|  | Dominant | TT + TC vs CC | 1.42 (1.02, 1.97) | 0.037* | 0.95 (0.63, 1.41) | 0.788 |
|  | Recessive | TT vs TC + CC | 1.31 (0.35, 4.94) | 0.692 | 0.81 (0.32, 2.04) | 0.657 |
|  | Additive | -- | 1.37 (1.01, 1.87) | 0.043* | 0.94 (0.68, 1.30) | 0.706 |
| **Smoking Status** | |  | **Yes** | | **No** | |
| rs12412320 | Allele | T vs G | 0.79 (0.59, 1.07) | 0.127 | 0.76 (0.60, 0.96) | 0.020* |
|  | Homozygous | TT vs GG | 0.53 (0.21, 1.34) | 0.182 | 0.73 (0.41, 1.31) | 0.286 |
|  | Heterozygous | TG vs GG | 0.83 (0.57, 1.22) | 0.349 | 0.58 (0.43, 0.79) | **0.001*** |
|  | Dominant | TT + TG vs GG | 0.80 (0.55, 1.15) | 0.221 | 0.60 (0.45, 0.81) | **0.001*** |
|  | Recessive | TT vs TG + GG | 0.57 (0.23, 1.42) | 0.226 | 0.89 (0.50, 1.58) | 0.698 |
|  | Additive | -- | 0.79 (0.58, 1.08) | 0.143 | 0.71 (0.56, 0.90) | **0.004*** |
| rs11245007 | Allele | T vs C | 1.25 (0.98, 1.59) | 0.071 | 1.05 (0.86, 1.28) | 0.610 |
|  | Homozygous | TT vs CC | 1.50 (0.90, 2.49) | 0.116 | 1.27 (0.86, 1.88) | 0.225 |
|  | Heterozygous | TC vs CC | 1.38 (0.90, 2.12) | 0.139 | 0.91 (0.66, 1.26) | 0.576 |
|  | Dominant | TT + TC vs CC | 1.42 (0.95, 2.12) | 0.091 | 1.02 (0.75, 1.38) | 0.910 |
|  | Recessive | TT vs TC + CC | 1.21 (0.80, 1.84) | 0.368 | 1.35 (0.96, 1.89) | 0.088 |
|  | Additive | -- | 1.23 (0.95, 1.58) | 0.110 | 1.11 (0.91, 1.35) | 0.294 |
| rs11245008 | Allele | T vs C | 1.20 (0.84, 1.71) | 0.318 | 1.13 (0.84, 1.52) | 0.411 |
|  | Homozygous | TT vs CC | 1.28 (0.25, 6.70) | 0.767 | 0.95 (0.40, 2.27) | 0.910 |
|  | Heterozygous | TC vs CC | 1.35 (0.89, 2.05) | 0.153 | 1.24 (0.87, 1.77) | 0.236 |
|  | Dominant | TT + TC vs CC | 1.35 (0.90, 2.03) | 0.149 | 1.20 (0.86, 1.69) | 0.288 |
|  | Recessive | TT vs TC + CC | 1.19 (0.23, 6.20) | 0.833 | 0.91 (0.38, 2.17) | 0.835 |
|  | Additive | -- | 1.30 (0.90, 1.91) | 0.164 | 1.13 (0.85, 1.51) | 0.407 |
| **Drinking Status** | |  | **Yes** | | **No** | |
| rs12412320 | Allele | T vs G | 0.90 (0.67, 1.21) | 0.471 | 0.70 (0.56, 0.89) | **0.003*** |
|  | Homozygous | TT vs GG | 0.61 (0.25, 1.51) | 0.286 | 0.66 (0.37, 1.20) | 0.177 |
|  | Heterozygous | TG vs GG | 0.91 (0.63, 1.32) | 0.618 | 0.57 (0.42, 0.77) | **< 0.001*** |
|  | Dominant | TT + TG vs GG | 0.87 (0.61, 1.25) | 0.449 | 0.58 (0.43, 0.78) | **< 0.001*** |
|  | Recessive | TT vs TG + GG | 0.63 (0.26, 1.54) | 0.314 | 0.83 (0.46, 1.48) | 0.522 |
|  | Additive | -- | 0.86 (0.63, 1.16) | 0.321 | 0.69 (0.54, 0.87) | **0.002*** |
| rs11245007 | Allele | T vs C | 1.08 (0.86, 1.37) | 0.499 | 1.17 (0.95, 1.42) | 0.143 |
|  | Homozygous | TT vs CC | 1.18 (0.73, 1.90) | 0.512 | 1.44 (0.96, 2.17) | 0.078 |
|  | Heterozygous | TC vs CC | 1.08 (0.70, 1.65) | 0.736 | 1.07 (0.77, 1.48) | 0.693 |
|  | Dominant | TT + TC vs CC | 1.11 (0.74, 1.66) | 0.608 | 1.17 (0.86, 1.59) | 0.314 |
|  | Recessive | TT vs TC + CC | 1.12 (0.76, 1.66) | 0.571 | 1.39 (0.97, 1.99) | 0.075 |
|  | Additive | -- | 1.08 (0.85, 1.38) | 0.511 | 1.19 (0.97, 1.45) | 0.098 |
| rs11245008 | Allele | T vs C | 1.09 (0.77, 1.55) | 0.633 | 1.20 (0.90, 1.62) | 0.220 |
|  | Homozygous | TT vs CC | 0.99 (0.25, 3.92) | 0.989 | 1.09 (0.43, 2.75) | 0.861 |
|  | Heterozygous | TC vs CC | 1.16 (0.76, 1.75) | 0.494 | 1.42 (0.99, 2.03) | 0.059 |
|  | Dominant | TT + TC vs CC | 1.14 (0.76, 1.72) | 0.515 | 1.38 (0.98, 1.94) | 0.069 |
|  | Recessive | TT vs TC + CC | 0.96 (0.24, 3.79) | 0.954 | 1.01 (0.40, 2.56) | 0.977 |
|  | Additive | -- | 1.11 (0.77, 1.60) | 0.566 | 1.27 (0.94, 1.71) | 0.116 |
| **BMI** |  |  | **> 24 kg/m^2^** | | **≤ 24 kg/m^2^** | |
| rs12412320 | Allele | T vs G | 0.96 (0.68, 1.36) | 0.811 | 0.75 (0.56, 1.00) | 0.050 |
|  | Homozygous | TT vs GG | 1.05 (0.44, 2.52) | 0.971 | 0.51 (0.23, 1.14) | 0.100 |
|  | Heterozygous | TG vs GG | 0.74 (0.46, 1.17) | 0.198 | 0.76 (0.52, 1.11) | 0.153 |
|  | Dominant | TT + TG vs GG | 0.78 (0.50, 1.21) | 0.268 | 0.72 (0.50, 1.04) | 0.079 |
|  | Recessive | TT vs TG + GG | 1.17 (0.49, 2.77) | 0.724 | 0.57 (0.26, 1.25) | 0.161 |
|  | Additive | -- | 0.88 (0.62, 1.24) | 0.458 | 0.74 (0.54, 1.00) | 0.047 |
| rs11245007 | Allele | T vs C | 1.10 (0.82, 1.48) | 0.525 | 0.95 (0.74, 1.22) | 0.680 |
|  | Homozygous | TT vs CC | 1.33 (0.73, 2.43) | 0.358 | 0.94 (0.56, 1.58) | 0.815 |
|  | Heterozygous | TC vs CC | 1.16 (0.72, 1.87) | 0.546 | 0.77 (0.50, 1.18) | 0.230 |
|  | Dominant | TT + TC vs CC | 1.21 (0.77, 1.89) | 0.468 | 0.82 (0.55, 1.22) | 0.330 |
|  | Recessive | TT vs TC + CC | 1.22 (0.72, 2.06) | 0.471 | 1.11 (0.71, 1.72) | 0.658 |
|  | Additive | -- | 1.15 (0.86, 1.55) | 0.348 | 0.95 (0.74, 1.23) | 0.713 |
| rs11245008 | Allele | T vs C | 0.93 (0.59, 1.48) | 0.763 | 1.09 (0.75, 1.57) | 0.649 |
|  | Homozygous | TT vs CC | 0.93 (0.20, 4.37) | 0.922 | 1.08 (0.38, 3.08) | 0.888 |
|  | Heterozygous | TC vs CC | 0.92 (0.54, 1.58) | 0.759 | 1.10 (0.70, 1.74) | 0.667 |
|  | Dominant | TT + TC vs CC | 0.92 (0.55- 1.56) | 0.751 | 1.10 (0.72, 1.69) | 0.659 |
|  | Recessive | TT vs TC + CC | 1.94 (0.20, 4.43) | 0.938 | 1.06 (0.37, 3.02) | 0.916 |
|  | Additive | -- | 0.93 (0.59, 1.47) | 0.763 | 1.08 (0.75, 1.54) | 0.686 |

*Abbreviation: SNP*: Single nucleotide polymorphism, OR: Odds ratio, 95% CI: 95% confidence interval.

*Note:* *p* values were calculated by logistic regression analysis without and with adjusted by sex, age, smoking, and drinking

* *p* < 0.05 was considered statistical significance.

Bold *p* means that the data is statistically significant after Bonferroni correction (*p* < 0.05/3).

**Table S2: Stratified analysis by clinical features for the association between *C10orf90* variants and the risk of colorectal cancer.**

| SNP | Model | Genotype | Stage (III/IV vs I/II) | | Lymph node metastasis (Yes vs No) | | Colon cancer | | Rectal cancer | |
| --- | --- | --- | --- | --- | --- | --- | --- | --- | --- | --- |
|  |  |  | OR (95% CI) | *p* | OR (95% CI) | *p* | OR (95% CI) | *p* | OR (95% CI) | *p* |
| rs12412320 | Allele | T vs G | 0.52 (0.35, 0.79) | **0.002*** | 0.71 (0.49, 1.04) | 0.075 | 0.78 (0.61, 0.98) | 0.034* | 0.81 (0.65, 1.01) | 0.062 |
|  | Homozygous | TT vs GG | 0.23 (0.08, 0.68) | **0.008*** | 0.38 (0.14, 1.02) | 0.054 | 0.59 (0.30, 1.15) | 0.119 | 0.93 (0.54, 1.61) | 0.790 |
|  | Heterozygous | TG vs GG | 0.59 (0.35, 1.01) | 0.054 | 0.82 (0.51, 1.33) | 0.419 | 0.75 (0.56, 1.01) | 0.059 | 0.69 (0.52, 0.91) | **0.009*** |
|  | Dominant | TT + TG vs GG | 0.51 (0.31, 0.85) | **0.010*** | 0.73 (0.47, 1.15) | 0.176 | 0.73 (0.55, 0.97) | 0.029* | 0.72 (0.55, 0.94) | **0.016*** |
|  | Recessive | TT vs TG + GG | 0.27 (0.09, 0.80) | 0.018* | 0.41 (0.16, 1.07) | 0.068 | 0.65 (0.34, 1.27) | 0.206 | 1.06 (0.62, 1.82) | 0.822 |
|  | Additive | -- | 0.53 (0.35, 0.80) | **0.003*** | 0.71 (0.50, 1.03) | 0.072 | 0.76 (0.60, 0.96) | 0.023* | 0.81 (0.65, 1.01) | 0.063 |
| rs11245007 | Allele | T vs C | 1.80 (1.27, 2.56) | **0.001*** | 1.18 (0.87, 1.59) | 0.294 | 1.07 (0.88, 1.30) | 0.516 | 1.15 (0.96, 1.39) | 0.125 |
|  | Homozygous | TT vs CC | 3.06 (1.50, 6.24) | **0.002*** | 1.42 (0.79, 2.58) | 0.244 | 1.17 (0.78, 1.73) | 0.448 | 1.33 (0.93, 1.89) | 0.120 |
|  | Heterozygous | TC vs CC | 1.36 (0.77, 2.42) | 0.288 | 0.98 (0.59, 1.63) | 0.949 | 1.19 (0.86, 1.65) | 0.282 | 0.96 (0.71, 1.31) | 0.804 |
|  | Dominant | TT + TC vs CC | 1.80 (1.05, 3.07) | 0.032* | 1.12 (0.70, 1.79) | 0.638 | 1.19 (0.87, 1.61) | 0.275 | 1.07 (0.81, 1.42) | 0.631 |
|  | Recessive | TT vs TC + CC | 2.53 (1.36, 4.73) | **0.003*** | 1.44 (0.87, 2.39) | 0.161 | 1.04 (0.74, 1.46) | 0.811 | 1.36 (1.00, 1.84) | 0.049 |
|  | Additive | -- | 1.71 (1.21, 2.40) | **0.002*** | 1.18 (0.88, 1.58) | 0.264 | 1.09 (0.89, 1.32) | 0.399 | 1.14 (0.95, 1.37) | 0.145 |
| rs11245008 | Allele | T vs C | 1.39 (0.84, 2.31) | 0.197 | 0.86 (0.56, 1.31) | 0.480 | 1.13 (0.85, 1.51) | 0.403 | 1.16 (0.89, 1.52) | 0.271 |
|  | Homozygous | TT vs CC | / | / | / | / | 0.83 (0.29, 2.35) | 0.730 | 1.15 (0.48, 2.79) | 0.755 |
|  | Heterozygous | TC vs CC | 1.27 (0.72, 2.22) | 0.409 | 0.72 (0.44, 1.16) | 0.178 | 1.24 (0.89, 1.73) | 0.202 | 1.19 (0.87, 1.63) | 0.272 |
|  | Dominant | TT + TC vs CC | 1.35 (0.77, 2.36) | 0.295 | 0.77 (0.48, 1.25) | 0.291 | 1.20 (0.87, 1.66) | 0.264 | 1.19 (0.88, 1.61) | 0.263 |
|  | Recessive | TT vs TC + CC | / | / | / | / | 0.80 (0.28, 2.25) | 0.267 | 1.11 (0.46, 2.68) | 0.817 |
|  | Additive | -- | 1.42 (0.83, 2.41) | 0.200 | 0.86 (0.55, 1.35) | 0.522 | 1.13 (0.85, 1.50) | 0.290 | 1.15 (0.88, 1.50) | 0.294 |

*Abbreviation: SNP*: Single nucleotide polymorphism, OR: Odds ratio, 95% CI: 95% confidence interval.

*Note:* *p* values were calculated by logistic regression analysis without and with adjusted by sex, age, smoking, and drinking

* *p* < 0.05 was considered statistical significance.

Bold *p* means that the data is statistically significant after Bonferroni correction (*p* < 0.05/3).
